# Prevalence of Urinary Tract Infection, Bacteremia, and Meningitis Among Febrile Infants Aged 8 to 60 Days With SARS-CoV-2

**DOI:** 10.1001/jamanetworkopen.2023.13354

**Published:** 2023-05-12

**Authors:** Paul L. Aronson, Jeffrey P. Louie, Ellen Kerns, Brittany Jennings, Sloane Magee, Marie E. Wang, Nisha Gupta, Christopher Kovaleski, Lauren M. McDaniel, Corrie E. McDaniel

**Affiliations:** 1 Section of Pediatric Emergency Medicine, Departments of Pediatrics and of Emergency Medicine, Yale School of Medicine, New Haven, Connecticut; 2 Division of Emergency Medicine, University of Minnesota, Masonic Children's Hospital, Minneapolis; 3 Division of Child Health Policy, Department of Pediatrics, University of Nebraska Medical Center, Omaha; 4 American Academy of Pediatrics, Itasca, Illinois; 5 Division of Hospital Medicine, Department of Pediatrics, Stanford University School of Medicine, Lucile Packard Children’s Hospital Stanford, Palo Alto, California; 6 Division of Hospital Medicine, Inova Children’s Hospital, Falls Church, Virginia; 7 Division of Pediatric Emergency Medicine, Department of Pediatrics, Inova Children’s Hospital, Falls Church, Virginia; 8 Division of Hospital Medicine, Department of Pediatrics, Johns Hopkins Children’s Center, Baltimore, Maryland; 9 Division of Hospital Medicine, Department of Pediatrics, University of Washington School of Medicine, Seattle Children’s Hospital, Seattle; 10 Now with Division of Hospital Medicine, Department of Pediatrics, University of Washington School of Medicine, Seattle Children’s Hospital, Seattle

## Abstract

**Importance:**

The prevalence of urinary tract infection (UTI), bacteremia, and bacterial meningitis in febrile infants with SARS-CoV-2 is largely unknown. Knowledge of the prevalence of these bacterial infections among febrile infants with SARS-CoV-2 can inform clinical decision-making.

**Objective:**

To describe the prevalence of UTI, bacteremia, and bacterial meningitis among febrile infants aged 8 to 60 days with SARS-CoV-2 vs without SARS-CoV-2.

**Design, Setting, and Participants:**

This multicenter cross-sectional study was conducted as part of a quality improvement initiative at 106 hospitals in the US and Canada. Participants included full-term, previously healthy, well-appearing infants aged 8 to 60 days without bronchiolitis and with a temperature of at least 38 °C who underwent SARS-CoV-2 testing in the emergency department or hospital between November 1, 2020, and October 31, 2022. Statistical analysis was performed from September 2022 to March 2023.

**Exposures:**

SARS-CoV-2 positivity and, for SARS-CoV-2–positive infants, the presence of normal vs abnormal inflammatory marker (IM) levels.

**Main Outcomes and Measures:**

Outcomes were ascertained by medical record review and included the prevalence of UTI, bacteremia without meningitis, and bacterial meningitis. The proportion of infants who were SARS-CoV-2 positive vs negative was calculated for each infection type, and stratified by age group and normal vs abnormal IMs.

**Results:**

Among 14 402 febrile infants with SARS-CoV-2 testing, 8413 (58.4%) were aged 29 to 60 days; 8143 (56.5%) were male; and 3753 (26.1%) tested positive. Compared with infants who tested negative, a lower proportion of infants who tested positive for SARS-CoV-2 had UTI (0.8% [95% CI, 0.5%-1.1%]) vs 7.6% [95% CI, 7.1%-8.1%]), bacteremia without meningitis (0.2% [95% CI, 0.1%-0.3%] vs 2.1% [95% CI, 1.8%-2.4%]), and bacterial meningitis (<0.1% [95% CI, 0%-0.2%] vs 0.5% [95% CI, 0.4%-0.6%]). Among infants aged 29 to 60 days who tested positive for SARS-CoV-2, 0.4% (95% CI, 0.2%-0.7%) had UTI, less than 0.1% (95% CI, 0%-0.2%) had bacteremia, and less than 0.1% (95% CI, 0%-0.1%) had meningitis. Among SARS-CoV-2–positive infants, a lower proportion of those with normal IMs had bacteremia and/or bacterial meningitis compared with those with abnormal IMs (<0.1% [0%-0.2%] vs 1.8% [0.6%-3.1%]).

**Conclusions and Relevance:**

The prevalence of UTI, bacteremia, and bacterial meningitis was lower for febrile infants who tested positive for SARS-CoV-2, particularly infants aged 29 to 60 days and those with normal IMs. These findings may help inform management of certain febrile infants who test positive for SARS-CoV-2.

## Key Points

**Question:**

What is the prevalence of urinary tract infection, bacteremia, and bacterial meningitis in febrile infants with SARS-CoV-2 aged 8 to 60 days?

**Findings:**

In this cross-sectional study of 14 402 febrile infants aged 8 to 60 days, the prevalence of urinary tract infection, bacteremia, and bacterial meningitis was low for infants who tested positive for SARS-CoV-2, particularly infants aged 29 days or older and those with normal inflammatory markers.

**Meaning:**

This study’s results suggest that the low prevalence of concomitant bacterial infections can potentially inform the evaluation and management of certain febrile infants with SARS-CoV-2.

## Teaser

This cross-sectional study examines the prevalence of urinary tract infection (UTI), bacteremia, and bacterial meningitis among febrile infants aged 8 to 60 days with SARS-CoV-2.

## Introduction

Of the nearly 200 000 febrile infants aged 60 days or younger who are evaluated in US emergency departments (EDs) each year,^1^ 7% to 10% have a urinary tract infection (UTI), 2% to 3% have bacteremia, and 0.5% to 1% have bacterial meningitis, the latter 2 infections collectively termed invasive bacterial infection (IBI).^2,3^ Most infants presenting with fever, however, have a viral infection, including many with upper respiratory tract infections such as respiratory syncytial virus, influenza, and rhinovirus.^4,5,6,7^ Febrile infants with documented respiratory viral infections have a lower prevalence of bacterial infection than infants who test negative. However, the risk of UTI and IBI varies by age and the type of virus.^4,5,6,7^ Additionally, the risk of UTI is still high enough among viral-positive infants to warrant routine urine testing in febrile infants who are 60 days or younger and the risk of IBI is not negligible, particularly in the youngest infants.^4,5,6,7^

Since March 2020, when COVID-19 was declared a pandemic, SARS-CoV-2 virus has become widespread.^8^ Fever is a common presenting symptom of SARS-CoV-2 in infants and children.^9,10^ Several studies have reported a lower to similar prevalence of UTI (0%-7%) among infants younger than 90 days of age who tested positive for SARS-CoV-2 compared with the baseline risk of UTI in febrile infants, with a variable prevalence of bacteremia (0%-14%) and no cases of bacterial meningitis.^11,12,13^ However, these studies were conducted in 2020 to early 2021, had small sample sizes (22 to 53 infants), and not all of the infants were febrile.^11,12,13,14^ Therefore, the prevalence of UTI and IBI in infants with fever and SARS-CoV-2 is largely unknown. Knowledge of the prevalence of UTI and IBI among febrile infants who test positive for SARS-CoV-2 can inform decision-making about invasive testing and disposition for these infants. To this end, our objective was to describe the prevalence of UTI, bacteremia, and bacterial meningitis among febrile infants aged 8 to 60 days with and without SARS-CoV-2 positivity in a large, geographically diverse sample. We also aimed to describe IBI prevalence among SARS-CoV-2–positive infants with normal vs abnormal inflammatory marker (IM) levels.

## Methods

### Study Design

We conducted a secondary analysis of a multicenter cross-sectional study as part of the American Academy of Pediatrics’ (AAP) Reducing Excessive Variability in Infant Sepsis Evaluation II (REVISE II) quality improvement initiative. The current study included 106 hospitals in the US and Canada from November 1, 2020, to October 31, 2022. We followed the Strengthening the Reporting of Observational Studies in Epidemiology (STROBE) reporting guideline. The Institutional Review Board of the AAP deemed this study exempt under exemption category 1 and did not require informed participant consent.

### Study Sample

We included full-term, previously healthy, well-appearing infants aged 8 to 60 days who were evaluated in the ED or hospitalized with a temperature of at least 38 °C (100.4 °F) in the ED, at home, or in a clinic in the preceding 24 hours,^15^ and who had SARS-CoV-2 testing obtained. We excluded infants who appeared ill, using a previously utilized algorithm to define ill appearance,^16,17^ those with complex medical conditions, diagnosis of bronchiolitis, or specific bacterial infections (eg, cellulitis), receipt of antibiotics or immunizations in the preceding 48 hours,^15^ and infants who were transferred from a referring hospital if laboratory tests were obtained prior to transfer. Exclusions were determined by medical record review and applied prior to data entry.

### Data Collection

Using medical record review, participating sites entered data on eligible infants on a monthly basis into a standardized, web-based data collection form. Sites were instructed to enter all eligible infants up to 20 per month, although individual sites could choose to enter more than 20. Not all sites entered data each month. Over the study period, 2.2% of site-months (the number of sites multiplied by the number of months with data entered) had exactly 20 infants entered and 4.3% had greater than 20 infants; a median (IQR) of 5 (2-10) infants were entered per site per month.

Data collected included demographics, laboratory testing results including urinalysis, AAP Clinical Practice Guideline (CPG)–recommended IMs (height of temperature, absolute neutrophil count [ANC], C-reactive protein [CRP], and procalcitonin [PCT]), blood, urine, and cerebrospinal fluid (CSF) cultures, and SARS-CoV-2 testing; whether the infant received a treatment course for UTI, bacteremia, or bacterial meningitis; whether the infant had a return visit within 7 days; and whether a UTI or IBI was diagnosed at the return visit. Race (using US census categories) and ethnicity were collected for the purpose of demonstrating the generalizability of our sample but not as analytic variables. For urinalysis and IMs, the data collection form listed the normal and abnormal values per the AAP CPG and participating sites chose the appropriate option. The abnormal values were defined as follows: (1) urinalysis with positive leukocyte esterase, positive nitrites, or greater than 5 white blood cells per high-powered field on microscopy^18^; (2) maximum temperature greater than 38.5 °C; (3) ANC greater than 4000 cells/mm^3^; (4) CRP greater than or equal to 2 mg/dL (to convert to mg/L, multiply by 10); and (5) PCT greater than 0.5 ng/mL.^15^

### Outcomes

Our primary outcomes were the prevalence of UTI, bacteremia without meningitis, and bacterial meningitis in SARS-CoV-2–positive vs SARS-CoV-2–negative infants, and the prevalence of IBI in SARS-CoV-2–positive infants with normal vs abnormal IMs. We stratified these outcomes by AAP CPG age groups (8 to 21 days, 22 to 28 days, and 29 to 60 days). A UTI was defined by the presence of a positive urinalysis with greater than or equal to 10 000 colony-forming units per high-powered field of a urinary pathogen on urine culture obtained by catheterization or suprapubic aspiration.^15^ Bacteremia was defined as the growth of an a priori defined pathogen on blood culture without growth of a pathogen in the CSF, and for which the infant received a treatment course for bacteremia. Bacterial meningitis was defined as growth of a pathogen on CSF culture, with or without concomitant bacteremia, and for which the infant received a treatment course for meningitis.^15,16^ For urine, blood, and CSF cultures with an a priori defined pathogen but for whom the site did not list that the infant received a treatment course for UTI, bacteremia, or meningitis, 2 investigators (P.L.A. and C.E.M.) reviewed the infant’s data and classified the organism as a pathogen or contaminant based on consensus, with a third investigator (M.E.W.) resolving discrepancies. Infants without urine, blood, and/or CSF cultures were presumed to not have UTI or IBI if not diagnosed within 7 days of discharge.

We also measured adherence to AAP CPG–recommended testing by age group for infants who tested positive for SARS-CoV-2 and those who tested negative. We defined adherence as urinalysis, blood culture, and CSF testing for infants aged 8 to 21 days and urinalysis, blood culture, and the AAP CPG–recommended combination of IMs for infants aged 22 to 60 days, defined as either (1) PCT and ANC or (2) temperature, ANC, and CRP.^15^

### Statistical Analysis

To calculate the prevalence of UTI, bacteremia without meningitis, and bacterial meningitis among infants with SARS-CoV-2 infection and those without, we used the denominator of all eligible febrile infants who had SARS-CoV-2 testing. We calculated 95% CIs for each infection type and stratified results by SARS CoV-2 positivity and AAP CPG age group.

To calculate the prevalence of IBI in infants with SARS-CoV-2 infection stratified by normal vs abnormal IMs, we limited the sample to infants who had IMs obtained. Infants were classified as having normal IMs if they received the AAP CPG–recommended combination of IMs (PCT and ANC or temperature and ANC and CRP) and had normal values for all IMs in the combination.^2,15^ Infants were classified as having abnormal IMs if any one of PCT, ANC, or CRP was abnormal. If PCT was not obtained, temperature was also considered an abnormal IM if elevated.^15^

We stratified results by AAP CPG age group. All analyses were conducted using R version 4.0.4 (R Project for Statistical Computing) from September 2022 to March 2023.

## Results

### Characteristics

Of the 16 296 febrile infants entered into the data collection system during the 24-month study period, 14 402 (88.4%) had SARS-CoV-2 testing. Among the 14 402 included in the study sample, 8143 infants (56.5%) were male; 3597 (25.0%) were Hispanic, 1349 (9.4%) were non-Hispanic Black, 5732 (39.8) were non-Hispanic White, 1919 (13.3%) were other race and ethnicity, 1805 (12.5%) had unknown race and ethnicity; 8413 (56.5%) were aged 29 to 60 days; and 3753 (26.1%) tested positive for SARS-CoV-2. Compared with infants who tested negative for SARS-CoV-2, a slightly larger proportion of infants who tested positive were aged 29 to 60 days. The proportion of SARS-CoV-2–positive infants evaluated at freestanding children’s hospitals, non–freestanding children’s hospitals, or general hospitals was similar (Table 1).

**Table 1.** Demographic Characteristics of Febrile Infants Aged 8 to 60 Days With and Without SARS-CoV-2

Characteristic	Infants, No. (%)		
	Total sample	SARS-CoV-2 positive	SARS-CoV-2 negative
Overall	14 402 (100.0)	3753 (26.1)	10 649 (73.9)
Age, d			
8-21	2657 (18.4)	412 (11.0)	2245 (21.1)
22-28	1904 (13.2)	431 (11.5)	1473 (13.8)
29-60	8413 (58.4)	2910 (77.5)	5503 (51.7)
Sex			
Female	6259 (43.5)	1627 (43.4)	4632 (43.5)
Male	8143 (56.5)	2126 (56.6)	6017 (56.5)
Race and ethnicity			
Hispanic	3597 (25.0)	1055 (28.1)	2542 (23.9)
Non-Hispanic Black	1349 (9.4)	390 (10.4)	959 (9.0)
Non-Hispanic White	5732 (39.8)	1327 (35.4)	4405 (41.4)
Other non-Hispanic^a^	1919 (13.3)	474 (12.6)	1445 (13.6)
Unknown	1805 (12.5)	507 (13.5)	1298 (12.2)
Region			
Northeast	2802 (19.5)	761 (20.3)	2041 (19.2)
South	5881 (40.8)	1471 (39.2)	4410 (41.4)
Midwest	2915 (20.2)	766 (20.4)	2149 (20.2)
West	2191 (15.2)	610 (16.3)	1581 (14.8)
Canada	613 (4.3)	145 (3.9)	468 (4.4)
Hospital type			
Freestanding children’s hospital	8460 (58.7)	2230 (59.4)	6230 (58.5)
Non–freestanding children’s hospital	4461 (31.0)	1131 (30.1)	3330 (31.3)
General hospital	1481 (10.3)	392 (10.4)	1089 (10.2)

^a^ Other non-Hispanic includes American Indian or Alaska Native, Asian, Native Hawaiian or other Pacific Islander, or more than 1 race.

### Adherence to AAP CPG–Recommended Testing

Compared with infants who tested negative for SARS-CoV-2, a lower proportion of infants who tested positive had the AAP CPG–recommended testing (77.4% [8244 of 10 649] vs 70.7% [2655 of 3753]), including blood culture performance (95.1% [10 126 of 10 649] vs 89.2% [3349 of 3753]). A lower proportion of SARS-CoV-2–positive infants aged 29 to 60 days had urinalyses, blood cultures, and IMs (Table 2). Of the 353 SARS-CoV-2–positive infants across age groups who did not receive a urinalysis or blood culture, 29 (8.2%) re-presented within 7 days; 1 (0.3%) was diagnosed with a UTI, and 0 with an IBI at the return visit.

**Table 2.** Adherence to AAP CPG–Recommended Testing by Age Group for Febrile Infants Aged 8 to 60 Days With SARS-CoV-2 vs Without

Variable	Infants, No. (%)	
	SARS-CoV-2 positive	SARS-CoV-2 negative
Overall		
Total No.	3753	10 649
Urinalysis	3421 (91.2)	10 119 (95.0)
Blood culture	3349 (89.2)	10 126 (95.1)
Inflammatory markers	2843 (75.6)	8501 (79.8)
Recommended testing^a^^,^^b^	2655 (70.7)	8244 (77.4)
Age 8-21 d		
Total No.	412	2245
Urinalysis	392 (95.2)	2115 (94.2)
Blood culture	404 (98.1)	2197 (97.9)
CSF testing	300 (72.8)	1815 (80.8)
Inflammatory markers	316 (76.7)	1664 (74.1)
Recommended testing^a^	290 (70.4)	1730 (77.1)
Age 22-28 d		
Total No.	431	1473
Urinalysis	410 (95.1)	1415 (96.1)
Blood culture	423 (98.1)	1445 (98.1)
Inflammatory markers	361 (83.8)	1226 (83.2)
Recommended testing^b^	347 (80.5)	1184 (80.4)
Age 29-60 d		
Total No.	2910	6931
Urinalysis	2619 (90.0)	6589 (95.1)
Blood culture	2522 (86.7)	6484 (93.6)
Inflammatory markers	2166 (74.4)	5611 (81.0)
Recommended testing^b^	2018 (69.3)	5330 (76.9)

Abbreviations: AAP, American Academy of Pediatrics; CPG, Clinical Practice Guideline; CSF, cerebrospinal fluid.

^a^ Recommended testing for infants aged 8 to 21 days defined as urinalysis, blood culture, and CSF testing.

^b^ Recommended testing for infants aged 22 to 60 days defined as urinalysis, blood culture, and the AAP CPG–recommended combination of IMs for infants aged 22 to 60 days, defined as either (1) PCT and ANC or (2) temperature, ANC, and CRP.

### Prevalence of UTI and IBI in Infants With SARS-CoV-2 vs Without

The prevalence of UTI, bacteremia without meningitis, and bacterial meningitis was substantially lower in SARS-CoV-2–positive infants than SARS-CoV-2–negative infants (Table 3). Overall, 38 of 3753 SARS-CoV-2–positive infants (1.0%; 95% CI, 0.7%-1.3%) had a concomitant UTI or an IBI, compared with 949 of 10 649 SARS-CoV-2–negative infants (8.9%; 95% CI, 8.4%-9.5%). Among infants with SARS-CoV-2 infection, 30 infants (0.8% [95% CI, 0.5%-1.1%]) had UTI, 7 infants (0.2% [95% CI, 0.1%-0.3%]) had bacteremia without meningitis, and 3 infants (<0.1% 95% [CI, 0%-0.2%]) had bacterial meningitis. Among infants who tested positive for SARS-CoV-2, the prevalence of UTI or IBI was 2.4% (95% CI, 0.9%-3.9%) in those aged 8 to 21 days, 3.0% (95% CI, 2.0%-4.0%) in those aged 22 to 28 days, and 0.5% (95% CI, 0.3%-0.8%) in those aged 29 to 60 days. Table 4 shows the demographics, IMs, and culture results of the 7 SARS-CoV-2–positive infants with bacteremia without meningitis and the 3 infants with bacterial meningitis. Five of the 7 infants with bacteremia had at least 1 abnormal laboratory IM, and all 3 infants with bacterial meningitis had multiple abnormal IMs. The 2 infants with normal laboratory IMs grew *Enterococcus* spp on blood culture, including 1 with concomitant growth of coagulase-negative *Staphylococcus*.

**Table 3.** Prevalence of UTI, Bacteremia, and Bacterial Meningitis in Febrile Infants Aged 8 to 60 Days With SARS-CoV-2 vs Without

Variable	Infants, No. (%) [95% CI]	
	SARS-CoV-2 positive	SARS-CoV-2 negative
Overall		
Total No.	3753	10 649
UTI^a^	30 (0.8) [0.5-1.1]	805 (7.6) [7.1-8.1]
Bacteremia without meningitis^b^	7 (0.2) [0.1-0.3]	221 (2.1) [1.8-2.4]
Bacterial meningitis	3 (0.1) [0-0.2]	53 (0.5) [0.4-0.6]
Any of the above^c^	38 (1.0) [0.7-1.3]	949 (8.9) [8.4-9.5]
Age 8-21 d		
Total No.	412	2245
UTI^a^	7 (1.7) [0.5-3.0]	276 (12.3) [10.9-13.7]
Bacteremia without meningitis^b^	3 (0.7) [0-1.6]	78 (3.5) [2.7-4.2]
Bacterial meningitis	1 (0.2) [0-0.7]	27 (1.2) [0.8-1.7]
Any of the above^c^	10 (2.4) [0.9-3.9]	332 (14.8) [13.3-16.3]
Age 22-28 d		
Total No.	431	1473
UTI^a^	10 (2.3) [1.4-3.2]	155 (10.5) [9.0-12.1]
Bacteremia without meningitis^b^	2 (0.5) [0.3-0.6]	48 (3.3) [2.4-4.2]
Bacterial meningitis	1 (0.2) [0.2-0.3]	7 (0.5) [0.1-0.8]
Any of the above^c^	13 (3.0) [2.0-4.0]	174 (11.8) [10.2-13.5]
Age 29-60 d		
Total No.	2910	6931
UTI^a^	13 (0.4) [0.2-0.7]	374 (5.4) [4.9-5.9]
Bacteremia without meningitis^b^	2 (<0.1) [0-0.2]	95 (1.4) [1.1-1.6]
Bacterial meningitis	1 (<0.1) [0-0.1]	19 (0.3) [0.2-0.4]
Any of the above^c^	15 (0.5) [0.3-0.8]	443 (6.4) [5.8-7.0]

Abbreviation: UTI, urinary tract infection.

^a^ UTI with or without bacteremia, not including UTI with concomitant bacterial meningitis.

^b^ Bacteremia with or without UTI, not including infants with bacterial meningitis.

^c^ An infant with UTI and concomitant bacteremia would be included in both the UTI and bacteremia without meningitis categories. The N for “Any of the above” may therefore not be the sum of the 3 infection types.

**Table 4.** Febrile Infants With SARS-CoV-2 Aged 8 to 60 Days With Bacteremia and/or Bacterial Meningitis

Age, d	Elevated temperature^a^	Abnormal urinalysis^b^	Elevated ANC^c^	Elevated CRP^d^	Elevated PCT^e^	Urine culture	Urine culture colony count	Blood culture	CSF culture
12	Yes	Yes	Yes	Yes	Not obtained	No growth	NA	*Salmonella* spp	*Salmonella* spp
14	No	No	Yes	No	No	No growth	NA	*Escherichia coli*	No growth
16	Yes	Yes	Yes	Not obtained	Not obtained	*Escherichia coli*	>100 000 cfu/hpf	*Escherichia coli*	No growth
17	Yes	Yes	Yes	Yes	Yes	*Escherichia coli*	>100 000 cfu/hpf	*Escherichia coli*	No growth
25	Yes	No	Yes	Yes	Yes	No growth	<10 000 cfu/hpf	*Escherichia coli*	*Escherichia coli*
27	No	No	No	No	Not obtained	*Enterococcus* spp and coagulase-negative *Staphylococcus*	<10 000 cfu/hpf	*Enterococcus* spp	No growth
27	Yes	No	Yes	Not obtained	Not obtained	No growth	NA	Group B *Streptococcus*	No growth
35	No	Yes	Yes	Yes	Yes	*Escherichia coli*	>100 000 cfu/hpf	*Escherichia coli*	*Escherichia coli*
37	Yes	Yes	Yes	Not obtained	Not obtained	*Escherichia coli*	>100 000 cfu/hpf	*Escherichia coli*	No growth
51	Yes	No	No	No	No	No growth	NA	*Enterococcus* spp and coagulase-negative *Staphylococcus*	No growth

Abbreviations: ANC, absolute neutrophil count; cfu, colony-forming unit; CRP, C-reactive protein; CSF, cerebrospinal fluid; hpf, high-powered field; NA, not applicable; PCT, procalcitonin.

^a^ Highest documented temperature greater than 38.5 °C (101.5 °F) in the emergency department, at home, or at a clinic in the preceding 24 hours.

^b^ Positive leukocyte esterase, positive nitrites, or greater than 5 white blood cells per hpf on microscopy.

^c^ ANC >4000/mm^3^.

^d^ CRP ≥20 mg/L or ≥2 mg/dL.

^e^ PCT >0.5 ng/mL.

### Prevalence of IBI in SARS-CoV-2–Positive Infants by Normal vs Abnormal IMs

Among the 2248 SARS-CoV-2–positive infants with normal IMs, 2 (<0.1%; 95% CI, 0%-0.2%) had an IBI, including 2 (<0.1%; 95% CI, 0%-0.2%) with bacteremia without meningitis and 0 with bacterial meningitis (Table 5). Among 436 SARS-CoV-2–positive infants with abnormal IMs, 8 (1.8%; 95% CI, 0.6%-3.1%) had an IBI, including 5 (1.1%; 95% CI, 0.2%-2.2%) with bacteremia without meningitis and 3 (0.7%; 95% CI, 0%-1.5%) with bacterial meningitis. Among SARS-CoV-2–positive infants aged 29 to 60 days, 1 infant (<0.1%; 95% CI, 0%-0.2%) with normal IMs and 2 infants (0.7%; 95% CI, 0%-1.8%) with abnormal IMs had IBIs.

**Table 5.** Prevalence of Bacteremia and Bacterial Meningitis Among Febrile Infants With SARS-CoV-2 Aged 8 to 60 Days With Normal vs Abnormal IMs

Age groups	Infants, No. (%) [95% CI]	
	SARS-CoV-2 positive and normal IMs^a^^,^^b^	SARS-CoV-2 positive and abnormal IMs^c^
Overall, No.	2248	436
Bacteremia without meningitis^d^	2 (<0.1) [0-0.2]	5 (1.1) [0.2-2.2]
Bacterial meningitis	0	3 (0.7) [0-1.5]
IBI^e^	2 (<0.1) [0-0.2]	8 (1.8) [0.6-3.1]
Age 8-21 d, No.	205	102
Bacteremia without meningitis^d^	0	3 (2.9) [0-6.2]
Bacterial meningitis	0	1 (1.0) [0-2.9]
IBI^e^	0	4 (3.9) [0.2-7.7]
Age 22-28 d, No.	297	63
Bacteremia without meningitis^d^	1 (0.3) [0-1.0]	1 (1.6) [0-4.7]
Bacterial meningitis	0	1 (1.6) [0-4.7]
IBI^e^	1 (0.3) [0-1.0]	2 (3.2) [0-7.5]
Age 29-60 d, No.	1746	271
Bacteremia without meningitis^d^	1 (<0.1) [0-0.2]	1 (0.4) [0-1.1]
Bacterial meningitis	0	1 (0.4) [0-1.1]
IBI^e^	1 (<0.1) [0-0.2]	2 (0.7) [0-1.8]

Abbreviations: IBI, invasive bacterial infection; IMs, inflammatory marker levels.

^a^ Infants were classified as having normal IMs if they received the AAP CPG–recommended combination of IMs (PCT and ANC or temperature and ANC and CRP) and had normal values for all IMs in the combination.

^b^ 95% CI not calculated when N = 0.

^c^ Infants were classified as having abnormal IMs if any one of PCT, ANC, or CRP was abnormal. If PCT was not obtained, temperature was also considered an abnormal IM if elevated.

^d^ Bacteremia with or without urinary tract infection, not including infants with bacterial meningitis.

^e^ IBI defined as bacteremia and/or bacterial meningitis.

## Discussion

In this large, geographically diverse convenience sample of 14 402 full-term, previously healthy, well-appearing febrile infants aged 8 to 60 days, infants with SARS-CoV-2 positivity had very low prevalence of UTI, bacteremia, and bacterial meningitis. The prevalence of bacteremia and bacterial meningitis was particularly low among SARS-CoV-2–positive infants aged 29 to 60 days and among infants with normal IMs across all 3 AAP CPG age groups. Given these findings, the integration of SARS-CoV-2 testing results into the risk stratification of febrile infants merits future study.

Although rapid viral polymerase chain reaction and multiplex respiratory viral testing has become increasingly prevalent and readily available,^19^ data on the prevalence of concomitant bacterial infection in the setting of a positive viral test are mixed for febrile infants. Multiple studies have shown a lower but still clinically important prevalence of UTI with viral positivity compared with viral negativity among febrile infants. These studies have also shown a lower prevalence of bacteremia and meningitis with viral positivity, but that IBI prevalence still remained high enough, particularly in infants aged 28 days or younger and with certain viruses (eg, rhinovirus), to warrant further testing for bacterial infection.^4,5,6,7^ Data on the prevalence of UTI and IBI among febrile infants with SARS-CoV-2 are even more limited. Several small studies conducted early in the pandemic reported a 0% to 7% UTI prevalence among SARS-CoV-2–positive infants younger than 90 days with a highly variable prevalence of bacteremia (0%-14%) and no cases of bacterial meningitis.^11,12,13,14^

The ongoing COVID-19 pandemic, however, has created a unique opportunity to assess the prevalence of concomitant bacterial infection as the majority of infants presenting for care had SARS-CoV-2 testing when testing became widely available; 88% in our convenience sample were tested. Overall, SARS-CoV-2–positive infants aged 8 to 60 days had a 0.8% prevalence of UTI, 0.2% prevalence of bacteremia, and less than 0.1% prevalence of bacterial meningitis, all of which were substantially lower than the prevalence of concomitant bacterial infection in SARS-CoV-2–negative infants. Although the prevalence of UTI and IBI may be low enough for some clinicians to consider deferring further testing for bacterial infection in SARS-CoV-2–positive infants, depending on their risk tolerance and practice setting, it is important to note that the prevalence of concomitant bacterial infection varied by age group and whether IMs were normal or abnormal. Specifically, SARS-CoV-2–positive infants aged 29 to 60 days had a 0.4% prevalence of UTI and less than 0.1% prevalence of bacteremia or bacterial meningitis. Additionally, only less than 0.1% of infants aged 8 to 60 days with normal IMs had bacteremia and 0% had bacterial meningitis. Of the 10 SARS-CoV-2–positive infants with an IBI, 8 had at least 1 abnormal laboratory IM, including all 3 infants with meningitis. The 2 infants with normal laboratory IMs grew *Enterococcus* spp on blood culture, which was treated as bacteremia though *Enterococcus* may also be a contaminant.^20^ Because clinicians might not have SARS-CoV-2 testing results immediately available for many febrile infants, clinicians may still use the AAP CPG–recommended IMs for risk stratification of infants aged 22 to 60 days.^15^ For febrile infants aged 8 to 21 days in whom SARS-CoV-2 is highly suspected, clinicians may consider use of IMs for risk stratification even though IMs are optional in this age group.^15^ SARS-CoV-2–positive infants aged 29 to 60 days with abnormal IMs still had a low though nonnegligible prevalence of bacteremia and bacterial meningitis. Our estimates of bacterial infection prevalence may inform shared decision-making between clinicians and parents about CSF testing, disposition, and empirical antimicrobial therapy for these older infants with abnormal IMs, an approach recommended by the AAP CPG.^15^

Our reported prevalence of bacterial infection, particularly UTI, is lower than the reported prevalence of concomitant UTI and IBI in febrile infants with respiratory viruses other than SARS-CoV-2.^4,5,6,7^ There are several potential explanations for this finding. First, during waves of the COVID-19 pandemic, presentation to the ED for other respiratory viruses was uncommon in children.^21,22^ This implied a lower rate of circulating respiratory viruses, except for SARS-CoV-2, with a resultant lower rate of baseline viral shedding. Therefore, it is possible that most infants aged 8 to 60 days who presented to the ED with fever found to be SARS-CoV-2 positive had active SARS-CoV-2 infection as the source of their fever.^23^ This would be in contrast to the potential asymptomatic viral shedding of other respiratory viruses, particularly rhinovirus.^24^ Second, our study included frequent testing for a specific virus, as 88% of infants underwent SARS-CoV-2 testing in our sample. It is possible that the low prevalence of concomitant bacterial infection with SARS-CoV-2 would be generalizable if we tested for other respiratory viruses more frequently in febrile infants. However, this routine testing approach for other respiratory viruses may not improve the accuracy of risk stratification algorithms for febrile infants, although further study is needed.^15^

In our sample, current practice deviated somewhat for SARS-CoV-2–positive febrile infants compared with SARS-CoV-2–negative infants in relation to the AAP CPG–recommended workup, with most of the difference in infants aged 29 to 60 days. Overall, approximately 71% of febrile infants with SARS-CoV-2 had a urinalysis, blood culture, and either the AAP CPG–recommended combination of IMs or CSF testing, based on age group. Future studies should address the effect of SARS-CoV-2 testing on clinical decision-making in relation to the AAP CPG–recommended workup.

### Limitations

Our study used a retrospective convenience sample, which has several limitations. First, although we included more than 14 000 febrile infants who received SARS-CoV-2 testing at 106 geographically diverse sites, we calculated UTI and IBI prevalence in a convenience sample of febrile infants, which may not reflect the true population prevalence. We do not have data on eligible infants not entered into the data collection system, potentially resulting in selection bias. Second, our study was descriptive, and we did not conduct an adjusted analysis to account for potential confounders or in-hospital clustering of UTIs or IBIs at larger hospitals. Third, although approximately 10% of SARS-CoV-2–positive infants did not have a urinalysis or blood culture obtained, none had a delayed diagnosis of IBI within 7 days. Fourth, assessment of clinical appearance retrospectively may not be reliable, although we used a previously used algorithm to determine ill appearance.^16,17^ Fifth, classification of pathogens vs contaminants may be limited retrospectively, although we classified infants using previously used definitions.^15,16^ Sixth, we excluded infants with bronchiolitis, which is infrequently caused by SARS-CoV-2.^25^ Seventh, our analysis of IBI prevalence among SARS-CoV-2–positive infants with normal IMs was limited to the subset of infants who had an AAP CPG–recommended combination of IMs obtained. However, more than 75% of infants in our sample had a full combination of IMs obtained. Eighth, we do not have details on SARS-CoV-2 testing protocols or which SARS-CoV-2 testing platforms were used at each site. Ninth, although a strength of our study is the 24-month study period that includes multiple waves of the COVID-19 pandemic, future studies should attempt to confirm our findings because the pandemic may have affected health care seeking behaviors of parents of febrile infants and SARS-CoV-2 prevalence continues to change over time.^23^

## Conclusions

In this large, geographically diverse sample of full-term, previously healthy, well-appearing febrile infants aged 8 to 60 days, the prevalence of UTI, bacteremia, and bacterial meningitis was lower for infants with SARS-CoV-2 positivity. The prevalence of UTI and IBI was particularly low among SARS-CoV-2–positive infants aged 29 to 60 days and in infants with normal IMs across all 3 AAP CPG age groups. Further prospective investigation is needed to evaluate how SARS-CoV-2 testing results affect the risk stratification and outcomes of febrile infants.

## Notes

Supplement 1.
Nonauthor Collaborators

Supplement 2.
Data Sharing Statement
