# Errors in Table 1 and Table 3

**DOI:** 10.1001/jamanetworkopen.2023.20575

**Published:** 2023-06-09

In the Original Investigation titled “Prevalence of Urinary Tract Infection, Bacteremia, and Meningitis Among Febrile Infants Aged 8 to 60 Days With SARS-CoV-2,”^1^ published May 12, 2023, there were errors in Table 1 and Table 3. In Table 1, the No. (%) of total infants aged 29 to 60 days should have been “9841 (68.3),” and the No. (%) of SARS-CoV-2–negative infants aged 29 to 60 days should have been “6931 (65.1).” In Table 3, the No. (%) [95% CI] of SARS-CoV-2–positive infants with bacterial meningitis should have been “3 (<0.1) [0-0.2].” This article has been corrected.^1^
