# Supplementary material for: Prevalence of Urinary Tract Infection, Bacteremia, and Meningitis Among Febrile Infants Aged 8 to 60 Days With SARS-CoV-2
Source: JAMA Netw Open. 2023 May 12;6(5):e2313354. doi: 10.1001/jamanetworkopen.2023.13354 (PMC10182434; doi:10.1001/jamanetworkopen.2023.13354)
Supplement: Supplement 1. — Nonauthor Collaborators [file jamanetwopen-e2313354-s001.pdf]

Supplemental Online Content: Nonauthor Collaborators
*First name, last name, and suffix (if applicable) are required and will appear in PubMed.

## *Group Name(s): AAP REVISE II QI Collaborative

| *First Name and Middle Initial(s) | *Last Name | *Suffix (eg, Jr, III) | Academic Degrees | Institution | Location (city, state/province, country) | Role or Contribution, eg, chair, principal investigator | Group (if more than 1 Group listed in the byline) and/or Subgroup (eg, Steering Committee) |
|---|---|---|---|---|---|---|---|
| Chisom | Agbim | | MD, MSHS | Children's Hospital Colorado, University of Colorado, Department of Pediatrics, Section of Emergency Medicine | Aurora, CO, USA | | |
| Owen | Amadasun | | MD, MBA | Hassenfeld Children's Hospital at New York University School of Medicine | New York, NY, USA | | |
| Nicholas | Beam | | MD | Spectrum Health/Helen Devos Children's Hospital | Grand Rapids, MI, USA | | |
| Bradley D. | Beamon | | MD | University of Mississippi Medical Center, Department of Pediatrics, | Jackson, MS, USA | | |
| Heather J. | Becker | | MD | Bridgeport Hospital, Department of Emergency Medicine | Bridgeport, CT, USA | | |
| Emma M. | Bedard | | Eng. BSE | Faculty of Medicine & Dentistry, University of Alberta, Department of Pediatrics | Edmonton, Alberta, Canada | | |
| Eric | Biondi | | MD, MSBA | Johns Hopkins University School of Medicine | Baltimore, MD, USA | | |
| Jenna | Boulet | | CCPA | Children's Hospital of Eastern Ontario | Ottawa, Ontariom, Canada | | |
| Amelia | Bray-Aschenbrenner | | MD | Washington University School of Medicine | St. Louis, MO, USA | | |
| Rona | Brooks | | MD | John Muir Medical Center Walnut Creek (Joint Venture with Stanford University School of Medicine Department of Pediatrics) | Walnut Creek, CA, USA | | |
| Clifford | Chen | | MD | UT Southwestern | Dallas, TX, USA | | |

Supplemental Online Content: Nonauthor Collaborators
*First name, last name, and suffix (if applicable) are required and will appear in PubMed.

| ***First Name and Middle Initial(s)** | ***Last Name** | ***Suffix (eg, Jr, III)** | Academic Degrees | Institution | Location (city, state/province, country) | Role or Contribution, eg, chair, principal investigator | Group (if more than 1 Group listed in the byline) and/or Subgroup (eg, Steering Committee) |
|---|---|---|---|---|---|---|---|
| Jacqueline B. | Corboy | | MD, MS | Northwestern Feinberg School of Medicine, Department of Pediatrics | Chicago, IL, USA | | |
| Nickolas | Dawlabani | | MD | Hovnanian Children's Hospital, Hackensack Meridian School of Medicine | Neptune City, NJ, USA | | |
| Guillermo | De Angulo | | MD, MSCR | Stanford Health Care - Lucile Packard Children's Hospital | Palo Alto, CA, USA | | |
| Senayit | Demie | | MD | Columbia University Irving Medical Center | New York, NY, USA | | |
| Jennifer | Drexler | | DO | Gundersen Health System | La Crosse, WI, USA | | |
| Nanette | Dudley | | MD | Spencer Fox Eccles School of Medicine, University of Utah | Salt Lake City, UT, USA | | |
| Andrew | Ellis | | MD | Mercy Children's Hospital St. Louis, Saint Louis University School of Medicine, Department of Pediatrics | Saint Louis, MO, USA | | |
| Kelsey | Fath | | MD | Dell Medical School, The University of Texas Austin | Austin, TX, USA | | |
| Alejandra | Frauenfelder | | MD | Nicklaus Children's Hospital | Miami, FL, USA | | |
| Jeff | Gill | | MD, MBA | University of California Riverside, Department of Pediatrics | Riverside, CA, USA | | |
| Thomas | Graf | | MD | Rainbow Babies and Children's Hospital, Division of Pediatric Hospital Medicine | Cleveland, OH, USA | | |
| Melissa | Grageda | | MD | Richmond University Medical Center | Staten Island, NY, USA | | |
| Heidi | Greening | | DO | Advocate Children's Hospital | Park Ridge, IL, USA | | |

Supplemental Online Content: Nonauthor Collaborators
*First name, last name, and suffix (if applicable) are required and will appear in PubMed.

| ***First Name and Middle Initial(s)** | ***Last Name** | ***Suffix (eg, Jr, III)** | Academic Degrees | Institution | Location (city, state/province, country) | Role or Contribution, eg, chair, principal investigator | Group (if more than 1 Group listed in the byline) and/or Subgroup (eg, Steering Committee) |
|---|---|---|---|---|---|---|---|
| Alison M | Griffin | | MD | Mass General Brigham Salem Hospital, Department of Pediatric Emergency Medicine | Salem, MA, USA | | |
| Amy | Groen | | DO, MHA | Blank Children's Hospital-UnityPoint Health | Des Moines, IA, USA | | |
| David T. | Guernsey III | | DO, MPH | Maimonides Medical Center | Brooklyn, NY, USA | | |
| Sarah | Gustafson | | MD | The Lundquist Institute at Harbor-UCLA Medical Center, David Geffen School of Medicine at University of California Los Angeles, Charles R Drew University of Medicine and Science | Los Angeles, CA, USA | | |
| W Caleb | Hancock | | MD | University of Oklahoma Health Sciences Center, Department of Pediatrics | Oklahoma City, OK, USA | | |
| Sujit | Iyer | | MD | Dell Children's Medical Center of Central Texas, UT Dell Medical School Austin | Austin, TX, USA | | |
| Patti Jo | Jaiyeola | | MD | St. Luke's University Health Network | Bethlehem, PA, USA | | |
| Aditi | Jayanth | | MD | NYC Health & Hospitals Kings County Medical Center | Brooklyn, NY, USA | | |
| Rebecca M. | Jennings | | MD | St. Charles Hospital | Bend, OR, USA | | |
| Svetlana S. | Kachan-Liu | | MD | Newton-Wellesley Hospital | Newton, MA, USA | | |
| Aditi | Kamat | | MD | Maria Fareri Children's Hospital at Westchester Medical Center | Valhalla, NY, USA | | |
| Isaac J. | Kennedy | | MD | West Virginia University Children's Hospital | Morgantown, WV, USA | | |
| Loay | Khateeb | | MD | Richmond University Medical Center, Department of Pediatrics | New York, NY, USA | | |

Supplemental Online Content: Nonauthor Collaborators
*First name, last name, and suffix (if applicable) are required and will appear in PubMed.

| ***First Name and Middle Initial(s)** | ***Last Name** | ***Suffix (eg, Jr, III)** | Academic Degrees | Institution | Location (city, state/province, country) | Role or Contribution, eg, chair, principal investigator | Group (if more than 1 Group listed in the byline) and/or Subgroup (eg, Steering Committee) |
|---|---|---|---|---|---|---|---|
| Ohmed | Khilji | | MD | Augusta University | Augusta, GA, USA | | |
| Elizabeth A. | Kleweno | | MD | St. Luke's Children’s Hospital | Boise, ID, USA | | |
| Anika | Kumar | | MD | Cleveland Clinic Children's | Cleveland, OH, USA | | |
| Stephanie | Kwon | | DO | Medical University of South Carolina, Department of Pediatrics | Charleston, SC, USA | | |
| Alicia | La | | DO | Huntington Hospital | Pasadena, CA, USA | | |
| Petra | Laeven-Sessions | | MD | Children’s Wisconsin | Neenah, WI, USA | | |
| Kaitlyn Phuong | Le | | MD | Sutter Medical Center | Sacramento, CA, USA | | |
| Rianna C. | Leazer | | MD | Children's Hospital of The King's Daughters, Division of Hospital Medicine | Norfolk, VA, USA | | |
| Kelly A. | Levasseur | | DO, MHA | Children’s Hospital of Michigan, Central Michigan University | Detroit, MI, USA | | |
| Emilee C. | Lewis | | MD | University of North Carolina School of Medicine, Department of Pediatrics | Chapel Hill, NC, USA | | |
| Walid M. | Maalouli | | MD | University of Minnesota, Department of Pediatrics, Division of Hospital Medicine | Minneapolis, MN, USA | | |
| Bonnie S. | Mackenzie | | MD | Lawrence & Memorial Hospital | New London, CT, USA | | |
| Kathleen E. | Mahoney | | MD | Medical College of Georgia at Augusta University, Department of Pediatrics | Augusta, GA, USA | | |
| Deborah M. | Margulis | | MD | Northwestern Lake Forest Hospital | Lake Forest, IL, USA | | |
| Lauren | Maskin | | MD | Children’s Hospital & Medical Center, University of Nebraska College of Medicine | Omaha, NE, USA | | |

Supplemental Online Content: Nonauthor Collaborators
*First name, last name, and suffix (if applicable) are required and will appear in PubMed.

| ***First Name and Middle Initial(s)** | ***Last Name** | ***Suffix (eg, Jr, III)** | Academic Degrees | Institution | Location (city, state/province, country) | Role or Contribution, eg, chair, principal investigator | Group (if more than 1 Group listed in the byline) and/or Subgroup (eg, Steering Committee) |
|---|---|---|---|---|---|---|---|
| Emily | McCarty | | MD | Kootenai Health Department of Pediatrics | Coeur d'Alene, ID, USA | | |
| Alexa N. | Monroy | | MD | Children's Hospital Los Angeles | Los Angeles, CA, USA | | |
| John M. | Morrison | | MD, PhD | Johns Hopkins All Children's Hospital, Division of Pediatric Hospital Medicine | St. Petersburg, FL, USA | | |
| Jennifer H. | Myszewski | | DO, PhD | State University of New York Upstate Medical University | Syracuse, NY, USA | | |
| Nicole L. | Nadeau | | MD | Massachusetts General Hospital, Department of Emergency Medicine | Boston, MA, USA | | |
| Suresh | Nagappan | | MD, MSPH | Cone Health/University of North Carolina, Department of Pediatrics | Greensboro, NC, USA | | |
| Kristen | Newcomer | | DO | Studer Family Children's Hospital at Sacred Heart Pensacola | Pensacola, FL, USA | | |
| Matthew C. | Nordstrom | | MD | University of California San Francisco | San Francisco, CA, USA | | |
| Diana | Nguyen | | DO | University of Texas Medical Branch | Galveston, TX, USA | | |
| Peter | O'Day | | MD | Columbus Regional Health | Columbus, IN, USA | | |
| Yuliya | Oumarbaeva-Malone | | MD | Children's National Medical Center | Washington, D.C., USA | | |
| Ursula | Parlin | | MD | UPMC Children's Hospital of Pittsburgh | Pittsburgh, PA, USA | | |
| Summer | Peters | | DO | Children's National Hospital | Washington, D.C., USA | | |
| Mary Jane | Piroutek | | MD | Children's Hospital of Orange County | Orange County, CA, USA | | |
| Renee | Quarrie | | M.B.B.S. | University of Maryland School of Medicine, Division of Pediatric Emergency Medicine | Baltimore, MD, USA | | |

Supplemental Online Content: Nonauthor Collaborators
*First name, last name, and suffix (if applicable) are required and will appear in PubMed.

| ***First Name and Middle Initial(s)** | ***Last Name** | ***Suffix (eg, Jr, III)** | Academic Degrees | Institution | Location (city, state/province, country) | Role or Contribution, eg, chair, principal investigator | Group (if more than 1 Group listed in the byline) and/or Subgroup (eg, Steering Committee) |
|---|---|---|---|---|---|---|---|
| Kerrilynn | Rice | | MD. MPH | Valley Children's Healthcare | Madera, CA, USA | | |
| Teresa M. | Romano | | MD | Lehigh Valley Reilly Children's Hospital | Allentown, PA, USA | | |
| Sahar N. | Rooholamini | | MD MPH | University of Washington, Department of Pediatrics | Seattle, WA, USA | | |
| Cynthia L. | Schroeder | | MD | St. Joseph's Children's Hospital | Tampa, FL, USA | | |
| Elizabeth K. | Segar | | MD | Medical College of Wisconsin | Milwaukee, WI, USA | | |
| Patrick | Seitzinger | | MD, MPH | University of Saskatchewan | Saskatoon, Saskatchewan, Canada | | |
| Suparna | Sharma | | MD | Lowell General Hospital, Department of Pediatrics | Lowell, MA, USA | | |
| Blair E. | Simpson | | MD | Cincinnati Children’s Hospital Medical Center, Division of Hospital Medicine, College of Medicine University of Cincinnati | Cincinnati, OH, USA | | |
| Prachi | Singh | | DO | University of California San Francisco, Department of Pediatrics, Division of Pediatric Infectious Diseases | Oakland, CA, USA | | |
| Yasmine | Sobeih | | MD | Sinai Hospital of Baltimore, Department of Pediatrics | Baltimore, MD, USA | | |
| Sakina | Sojar | | MD | Hasbro Children's Hospital, Division of Pediatric Emergency Medicine, Warren Alpert Medical School of Brown University | Providence, RI, USA | | |
| Karthik | Srinivasan | | MD | Cook Children’s Medical Center | Fort Worth, TX, USA | | |
| Emily C. | Sterrett | | MD, MS | Duke University, Department of Pediatrics | Durham, NC, USA | | |
| Mary Elizabeth | Swift-Taylor | | MD | Providence St Vincent Medical Center | Portland, OR, USA | | |

Supplemental Online Content: Nonauthor Collaborators
*First name, last name, and suffix (if applicable) are required and will appear in PubMed.

| ***First Name and Middle Initial(s)** | ***Last Name** | ***Suffix (eg, Jr, III)** | Academic Degrees | Institution | Location (city, state/province, country) | Role or Contribution, eg, chair, principal investigator | Group (if more than 1 Group listed in the byline) and/or Subgroup (eg, Steering Committee) |
|---|---|---|---|---|---|---|---|
| Sara | Szkola | | MD | Stanford University School of Medicine, Department of Pediatrics, Stanford Health Care Valleycare | Pleasanton, CA, USA | | |
| Jennifer | Thull-Freedman | | MD, MSc | University of Calgary Cumming School of Medicine, Departments of Pediatrics and Emergency Medicine | Calgary, Alberta, Canada | | |
| Irina | Topoz | | MD | Children's Hospital Colorado, Section of Emergency Medicine | Aurora, CO, USA | | |
| Tatyana | Vayngortin | | MD | Rady Children's Hospital San Diego; Department of Pediatrics, Division of Emergency Medicine, University of California San Diego | San Diego, CA, USA | | |
| Christopher | Veit | | MD, MHPE | Children's Hospital Kansas City | Kansas City, MO, USA | | |
| Yenimar | Ventura-Polanco | | MD | University of Florida College of Medicine- Jacksonville | Jacksonville, FL, USA | | |
| Ashley | Wallace Wu | | MD | Johns Hopkins School of Medicine, Department of Pediatrics | Baltimore, MD, USA | | |
| Lindsay | Weiss | | MD | Joe DiMaggio Children's Hospital | Hollywood, FL, USA | | |
| Erika K. | Wong | | MD | California Pacific Medical Center - Stanford University Department of Pediatrics | San Francisco, CA, USA | | |
| Sara | Zafar | | DO | Texas Children's Hospital, Baylor College of Medicine, Department of Pediatrics | Houston, TX, USA | | |
