# Supplementary material for: Prevalence of Urinary Tract Infection, Bacteremia, and Meningitis Among Febrile Infants Aged 8 to 60 Days With SARS-CoV-2
Source: JAMA Netw Open. 2023 May 12;6(5):e2313354. doi: 10.1001/jamanetworkopen.2023.13354 (PMC10182434; doi:10.1001/jamanetworkopen.2023.13354)
Supplement: Supplement 2. — Data Sharing Statement [file jamanetwopen-e2313354-s002.pdf]

Data Sharing Statement

Aronson. Prevalence of Urinary Tract Infection, Bacteremia, and Meningitis Among Febrile Infants Aged 8 to 60 Days With SARS-CoV-2. *JAMA Netw Open*. Published May 12, 2023. doi:10.1001/jamanetworkopen.2023.13354

Data
Data available: No

Additional Information
Explanation for why data not available: The hospitals participating in this quality improvement collaborative did not give written consent for their data to be shared publicly, so supporting data are not available.
